# Ethnomedicine of the Kagera Region, north western Tanzania. Part 2: The medicinal plants used in Katoro Ward, Bukoba District

**DOI:** 10.1186/1746-4269-6-19

**Published:** 2010-07-22

**Authors:** Mainen J Moshi, Donald F Otieno, Pamela K Mbabazi, Anke Weisheit

**Affiliations:** 1 Department of Biological and Preclinical Studies, Institute of Traditional Medicine, MUHAS, P.O. Box 65001, Dar es Salaam, Tanzania; 2 Department of Biological Sciences, Moi University, Eldoret, Kenya; 3 Faculty of Development Studies, Mbarara University of Science and Technology (MUST), P.O. Box 1410, Mbarara, Uganda

## Abstract

**Background:**

The Kagera region of north western Tanzania has a rich culture of traditional medicine use and practices. The dynamic inter-ethnic interactions of different people from the surrounding countries constitute a rich reservoir of herbal based healing practices. This study, the second on an ongoing series, reports on the medicinal plant species used in Katoro ward, Bukoba District, and tries to use the literature to establish proof of the therapeutic claims.

**Methodology:**

Ethnomedical information was collected using Semi-structured interviews in Kyamlaile and Kashaba villages of Katoro, and in roadside bushes on the way from Katoro to Bukoba through Kyaka. Data collected included the common/local names of the plants, parts used, the diseases treated, methods of preparation, dosage, frequency and duration of treatments. Information on toxicity and antidote were also collected. Literature was consulted to get corroborative information on similar ethnomedical claims and proven biological activities of the plants.

**Results:**

Thirty three (33) plant species for treatement of 13 different disease categories were documented. The most frequently treated diseases were those categorized as specific diseases/conditions (23.8% of all remedies) while eye diseases were the least treated using medicinal plants (1.5% of all remedies). Literature reports support 47% of the claims including proven anti-malarial, anti-microbial and anti-inflammatory activity or similar ethnomedical uses. Leaves were the most frequently used plant part (20 species) followed by roots (13 species) while making of decoctions, pounding, squeezing, making infusions, burning and grinding to powder were the most common methods used to prepare a majority of the therapies.

**Conclusion:**

Therapeutic claims made on plants used in traditional medicine in Katoro ward of Bukoba district are well supported by literature, with 47% of the claims having already been reported. This study further enhances the validity of plants used in traditional medicine in this region as resources that can be relied on to provide effective, accessible and affordable basic healthcare to the local communities. The plants documented also have the potential of being used in drug development and on farm domestication initiatives.

## Introduction

The Kagera region has a magnificent culture of herbalism. While the Haya tribe dominates the region, there is a lot of knowledge exchange with the neighboring tribes like the Rukiga and Banyankore of Uganda, the Tutsi and the Hutu of Rwanda and Burundi who have all intermarried overtime bringing together an impressive culture of herbal centered traditional medicine [1]. Thus when one talks to people belonging to different tribes within Kagera, regardless of their education status or age, what one hears is an impressive account of herbal therapies that have been used successfully to treat different diseases.

The first in the series of investigations on plants used in traditional medicine in the Kagera region reported on the plants used in Bugabo Ward [2]. This second part of the series provides a glimpse into the plants used in traditional medicine by Issack Kato and two of his colleagues; Maruzuku Mazimpaka and Hajat Nuria Kyejo, all who are traditional healers practicing in Katoro Ward. This study therefore adds to the continuing efforts to document [2], evaluate for biological activity [3-5], and identify how plant genetic resources in the Kagera region can be mainstreamed into the social and economic development of the local people, for example, through on-farm cultivation and the development of marketable medicinal plant products. The study is an ethnomedical documentation of medicinal plants in Katoro Ward of, Bukoba district, north western Tanzania.

## Methodology

### Description of the study site

**Figure 1:**
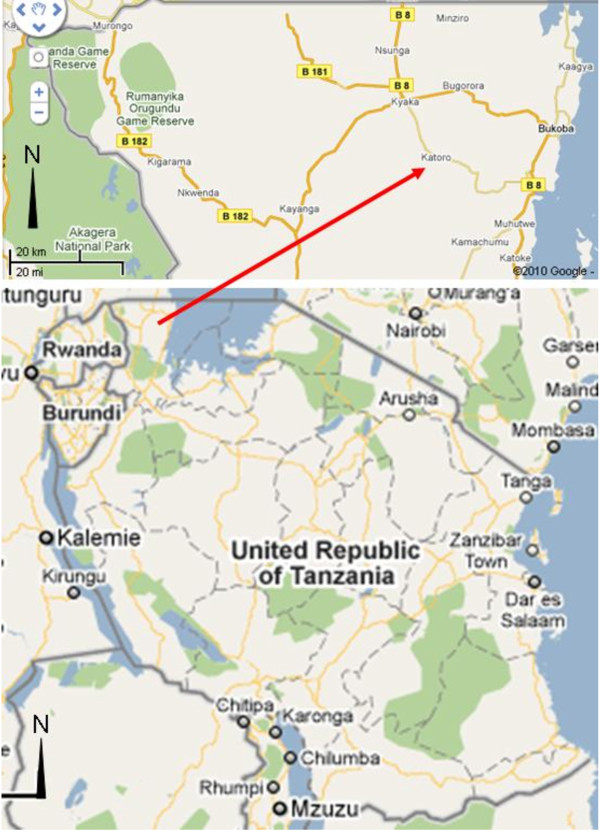
**Map showing the study site at Katoro ward, Bukoba District (Source: Google Maps 2010)**.

Katoro is a ward within Bukoba district and lies on the south west of Bukoba town and situated at 1° 23' 59'' South, 31° 30' 1'' East (Figure 1). Like the rest of the Bukoba district, the Katoro ward has good rainfall and good vegetation cover that provides abundant resources for traditional medicines.

### The Ethnobotanical visit and documentation of plant information

Independently, the research team established contact with three informants (Issack Kato, Maruzuku Mazimpaka and Hajat Nuria Kyejo) who practice traditional medicine in Kashaba village in Katoro. A field visit was, thereafter, made to the area between 28^th ^February and 2^nd ^March, 2008. During this visit ethnomedical information was collected using semi-structured interviews [6] as the team walked, accompanied by the informants, through the banana farms, roadside and surrounding bushes and thickets of Kyamlaile and Kashaba villages. Voucher specimens were made for all plants collected and these were subsequently identified by Mr. Selemani Haji of the Department of Botany, University of Dar es Salaam. Duplicate vouchers are kept at the Herbaria of the Botany Department, University of Dar es Salaam, and that of the Institute of Traditional Medicine, Muhimbili University of Health and Allied Sciences.

### Literature survey to establish proof of claims

Literature information was retrieved from the NAPRALERT data base at the School of Pharmacy, University of Illinois at Chicago. The strength of information obtained from the informants was evaluated based on its agreement with similar therapeutic claims in literature from elsewhere or evidence in literature of laboratory results that support the claims.

## Results

### Medicinal Plant diversity

A total of 33 plant species belonging to 31 genera and 19 plant families were documented (See additional file 1)). The largest proportion of medicinal plants collected belonged to the family Asteraceae (21%), followed by Fabaceae (12.1% each) and Euphorbiaceae (9.0%). The main source of these plants in terms of number of species were trees (30.3% of the total number of species) followed by shrubs (39.4%) and herbs (21.2%). The remaining 9.09% were climber herbs and shrubs.

### Diseases treated

A wide variety of medical conditions were treated using remedies made from medicinal plants. Most of the plants used had more than a single therapeutic use. For example, *Draceana steudneri *was used for treating fibroids, splenomegaly and asthma. On the other hand, many diseases were also treated using a wide range of plants. Malaria for example, was treated using *Senna alata*, *Clerodendrum myricoides*, *Dalbergia nitidula*, *Eriosema psoraleoides*, *Hygrophylla auriculata*, *Rhus vulgaris *and *Vernonia amygdalina*. The most frequent ailments treated with medicinal plants were those categorized here as specific diseases/conditions (Table 1) comprising conditions like malaria, dysentery, cancer, yellow fever etc. and were treated using the largest number of remedies (23.8% of all remedies). On the lower end, 1.5% of the remedies were used to treat eye diseases, 2.98% cardiovascular and circulatory diseases (e.g. anemia), 4.47% respiratory tract infections (e.g. chest pains) and 4.47% skeletal muscular problems (e.g. body spasms). Reproductive problems like difficulties to conceive and low libido were treated using 14.2% of all the remedies used.

**Table 1:** Number of plant species used to treat diseases within different disease categories (The disease categories were adopted from Ssegawa and Kasenene, 2007[40])

Disease Category	Number of plants
Cardiovascular and circulatory	2
Gastro-intestinal diseases	6
Respiratory tract infections	3
Eye diseases	1
Female genital system	4
Skeletal muscular system	3
Skin diseases and subcutaneous tissue	7
Infectious diseases	10
Child hood diseases and conditions	2
Specific diseases and conditions	16

### Plant parts used

The plant parts used for making herbal preparations were the roots, leaves, stem bark, root bark, pods and other aerial parts. The leaves were the most frequently used (20 species) followed by the roots (13 species), and stem bark and other aerial parts (each 4 species). Other parts like the pods were also used, for example in *Kigelia africana *and the root bark in *Parinari curatellifolia*, but rarely.

### Herbal medicines and their preparation

Mono therapies based on preparations made from a single plant were the most dominant, although many remedies where more than one plant was used were also common. Those that involved the use of two species included, for example, the boiling of *Carissa tomentosa *roots with the bark of *Elaedendron buchananii *or powders of the two being mixed and taken with tea or mixed with roots of *Tragia furialis *for the treatment of hernia, backache or taken as an aphrodisiac. Others included a decoction made from boiling the roots of *Combretum collinum *and *Rhus vulgaris *being drunk for the treatment of dysentery while another made from boiling the leaves of *Dalbergia nitidula *with the stem bark of *Sapium ellipticum *was used to treat malaria. Fresh leaves of *Dichrocephala integrifolia *were pounded with the leaves of *Ageratum conyzoides *and the juice squeezed out and applied to the eyes as an eye drop while for the treatment of indigestion, the leaves of *Hoslundia opposita *were mixed with the leaves of *Ocimum basilicum*, boiled and the decoction drunk. A decoction made from boiling the leaves of *Pappea capensis *with those of *Vernonia brachycalyx *was drunk for the treatment of backaches and to treat chickenpox, the leaves of *Rhus natalensis *mixed with those of *Vernonia amygdalina *were boiled and the decoction drunk. Treatments that involved the use of three or more plants in combination included, for example, the pounding of the roots and/or leaves of *Desmodium salicifolium*, *Elaeodendron buchananii *and *Tragia furialis *then boiling and taking the decoction as an aphrodisiac. Others included the treatment of skin rashes and joint pains and relieving of feet from burning sensations by applying the root or stem bark powder of *Maytenus senegalensis *mixed with the root powders of *Rauvolfia vomitora*, *Parinari curatellifolia *and *Ozoroa insignis *in a fat base. The treatment of yellow fever involved pounding and boiling the leaves of *Trema orientalis *with those of *Combretum collinum *and *Erythrina abbysinica *and taking the decoction. Backache was also treated using a decoction prepared from a combination of four different species. The decoction was made by boiling the root powder of *Tragia furialis *mixed with that of *Elaeodendron buchananii *or *Spathodea campanulata *and *Carisa spinarum *and then drunk or the powders were simply mixed with water and taken. A second treatment of malaria involved taking a decoction made from the leaves and/or roots of *Vernonia amygdalina *mixed with the stem bark of *Rhus natalensis *and the leaves of *Dalbergia nitidula*, *Desmodium salicifolium *and *Eriosema psoraleoides*. The most common methods used to prepare most of the therapies were making of decoctions (46.4%), pounding (14.2%), squeezing (10.7%), making infusions (8.9%), burning (7.1%) and grinding to powder (5.4%).

### Literature based proof of traditional healers' claims

Out of all the plants used by traditional healers in Katoro, the uses of 47% of them (16 out of 34 species) are supported by reports of similar uses or proven biological activity in the literature. There were no reports of toxicity for any of the species except for *Ageratum conyzoides *reported to have caused toxicity to sheep [7]. The plants whose therapeutic claims are well supported by the literature include *Ageratum conyzoides *[8-10], *Bidens pilosa *[11-14], *Boerhavia diffusa *[15], *Capparis tomentosa *[16], *Cassia alata *[17-19], *Clerodendrum myricoides *[20,21]. Others are *Combretum collinum *[22], *Dichrocephala integrifolia *[23], *Flueggea virosa *[24,25], *Hoslundia opposita *[26], *Jatropha curcas *[27,28], *Lantana camara *[29-31], *Melanthera scandens *[32], *Microglossa pyrifolia *[33,34], *Rubia cordifolia *[35-37] and *Vernonia amygdalina *[32,38,39].

## Discussion

This is the second of an ongoing series to document plants that are used in Kagera region, northwestern Tanzania, as traditional medicines. The plants that have been documented from Katoro are relatively few compared to the rich plant diversity that is known to be in the Kagera region [2]. However, the proportion of claims made by traditional healers in Katoro concerning some of the plants documented in this study and which are supported by literature evidence of proven biological activity or similar ethnomedical uses elsewhere is remarkable. Thus therapeutic claims made concerning; *Ageratum conyzoides*, *Bidens pilosa, Boerhavia diffusa*, *Capparis tomentosa*, *Cassia alata*, *Clerodendrum myricoides*, *Combretum collinum*, *Dichrocephala integrifolia*, *Flueggea virosa*, *Hoslundia opposita Jatropha curcas*, *Lantana camara*, *Melanthera scandens*, *Microglossa pyrifolia*, *Rubia cordifolia *and *Vernonia amygdalina *can be taken to be credible, given that these plants either have identical uses elsewhere or their biological activities have been proven. It has been suggested that the identical use of a medicinal plant by different people from different areas is often considered to be a good and reliable indicator of the plants curative properties [40].

The Kagera region is one place in Tanzania where there is a remarkable interchange of culture by ethnic groups from different countries e.g. the Rukiga and Banyankore of Uganda, the Tutsi and the Hutu of Rwanda and Burundi all who have intermarried over time bringing together an impressive culture of herbal centered traditional medicine [1]. This culture is indeed entrenched among the different ethnic groups in Kagera, and unlike other parts of Tanzania, people from all walks of life value traditional medicine, including even the well educated, who in other places would not so proudly talk of the benefits of traditional medicines.

Some plants previously documented in Kagera and used for the treatment of bacterial infections and wound healing [2] have been found to have antibacterial properties [3,5] and results from brine shrimp toxicity tests also suggest that they have low toxicity [4]. This goes to show that Kagera region, within which Katoro falls, has a repository of plants that can be relied upon for the treatment of various illnesses that the local communities have to deal with now and again.

## Conclusion

This study shows that the therapeutic claims made on plants used in traditional medicine in Katoro ward of Bukoba district are credible given that 47% of the claims are well supported by the literature. It also enhances the validity of the plants as resources that can be relied on to provide effective and affordable healthcare to the local communities. The plants documented in this study thus also have the potential of being used in drug development and on farm domestication/cultivation initiatives.

## Competing interests

The authors have no competing interests in the project, and share the aspirations of the local people of Katoro ward to bring good healthcare services to their community.

## Authors' contributions

MJM, DFO, AW, PKM, carried out the design of the study, which is being implemented in Kenya, Tanzania and Uganda. MJM interviewed traditional healers in Bukoba Rural District, compiled the information which was subsequently synthesized by MJM, AW and DFO to this final manuscript. All authors read, revised and approved the final manuscript.

## Supplementary Material

Additional file 1 **Medicinal plants used in Katoro ward; Bukoba District**. The file contains a list of medicinal plant species, their uses, parts used and methods of preparation, together with information from the literature supporting the traditional therapeutic claims. Click here for file
