# Supplementary material for: Ethnomedicine of the Kagera Region, north western Tanzania. Part 2: The medicinal plants used in Katoro Ward, Bukoba District
Source: J Ethnobiol Ethnomed. 2010 Jul 22;6:19. doi: 10.1186/1746-4269-6-19 (PMC3224970; doi:10.1186/1746-4269-6-19)
Supplement: Additional file 1 — Medicinal plants used in Katoro ward; Bukoba District. The file contains a list of medicinal plant species, their uses, parts used and methods of preparation, together with information from the literature supporting the traditional therapeutic claims. [file 1746-4269-6-19-S1.PDF]

Additional file 1. Medicinal plants used in Katoro ward, Bukoba District

Botanical Name (Family), [Voucher Number]	Vernacular Name	Life Form	Uses	Used Part	Method of Preparation/administration	Support of claims from the literature
Ageratum conyzoides L. (ASTERACEAE) [MJM 3211]	Katabataba	H	Cough remedy, constipation/ Peptic ulcers and fibroids with difficulties to conceive	R, L	Roots chewed fresh as an antacid and antiseptic while leaves boiled and decoction taken as tea.	Although it is reported to cause toxicity in sheep leading to death [7], hot water decoction of leaves is used as a cold remedy [8], and to prevent the birth of a quick succession of babies [9]. It is also used as a remedy for constipation [10]
Bidens pilosa L. (ASTERACEAE) [MJM 3207]	Kakurura	H	Anaemia; wounds HIV/AIDS and to aid in conception	L	Leaves are eaten as vegetable for treatment of anaemia and also squeezed and the exudates are applied to wounds as iodine tincture. The leaves are softened by wrapping the them in banana leaves and placing them under ash	Aerial parts and leaves used as antiseptic properties, is used for dressing wounds and aerial parts infusion has antioxidant and immunostimulant activity [11, 12, 13, 14].
Boerhavia diffusa L. (NYCTAGINACEAE) [MJM 3189]	Kimotoka	H	Peptic ulcers	AP	Aerial parts pounded, soaked in water and decanted. Half to one glass is then taken three times a day. The dry powder can also be taken with water or in tea.	Hot water extract of the dried leaves is used to treat dyspepsis ,a mechanism possibly mediated through cholinergic and histaminergic pathways [15]
Capparis tomentosa Lam. (CAPPARACEAE) [MJM 3215]	Ruvulinganga	CLS	Chest pains, loss of speech, skin diseases and burning sensation of the skin	R	Dry roots are either boiled with water or made into powder and taken with tea.	A decoction of the dried roots is used as a remedy for cough and colds [16].

Carissa spinarum L. Mantas (APOCYNACEAE) [MJM 3191]	Omukuyomonza	S	Hernia, aphrodisiac, backache	R	Roots are boiled with the bark of *Elaedendron buchanannii* or a powder made from pounding the roots of this species mixed with the bark of *Elaedendron buchanannii* is taken with tea. They can also be mixed with *Tragia Furialis* Boj roots	
Senna alata L. (CAESALPINIACEAE) [MJM 3216]	Mgalula	S	Malaria and dysentery	L, R	To treat Malaria the leaves are pounded and and boiled with water and decoction drank. For dysentery the roots are boiled and decoction drank	A decoction of the plant is used for treatment of diarrhea , which corroborates with antibacterial activity against *Salmonella typhi*, *Shigella boydi* , and a decoction of the leaves is used for treatment of fever and malaria [17, 18, 19].
Clerodendrum myricoides Bak (VERBENACEAE) [MJM 3198]	Mkuzanyana	S	Malaria, febrile convulsions, abdominal colics	SB, R	The stem bark/root boiled with water and half teaspoonful administered	Its extracts have antiplasmodial against Plasmodium falciparum and antidiarrheal activity [20, 21]
Combretum collinum Fresen (COMBRETACEAE) [MJM 3194]	Mkoyoyo	T	Diarrhea, dysentery, antispasmodic	R	The roots are boiled with water and decoction taken to treat diarrhea. In the treatment of dysentery it is combined with *Rhus vulgaris* Meikle roots	A methanol extract exhibited both antibacterial activity against *Psedomonas aeruginosa* and antifungal activity against *Candida albicans* and *Aspergillus niger* [22]
Craterispermum schweinfirthii Hiern. (RUBIACEAE) [MJM 3217]	Omushekera	T	Yellow fever; source of sugar	L, SB	The leaves/stem bark are dried and ground into a powder. The powder is then given to the patient to lick	
Dalbergia nitidula Bak. (FABACEAE) [MJM 3214]	Ruguma	T	Malaria	L	Leaves are boiled with the stem bark of *Sapium ellipticum*	

Desmodium salicifolium Poir. DC. (FABACEAE) [MJM 3201]	Mkongorana	S	Aphrodisiac	R, L	The roots/leaves are pounded together with *Elaeodendron buchananii* and *Tragia furialis*, boiled in water and a glass taken daily.	
Dichrocephala integrifolia (L.f.)Kuntze (ASTERACEAE) [MJM 3188]	Ibuza	H	Mouth ulcers, eye infections	L	The leaves are pound together with *Ageratum conyzoides*. Fresh leaves are pounded, juice squeezed out and applied as eye drops three times a day for three days.	Used to heal sores and stomach ulcers, which is probably associated with anti-staphylococcal activity [23]
Dracaena steudneri Engl. (AGAVACEAE) [MJM 3206]	Mgorogoro	T	Hernia, splenomegaly, asthma, chest problems in children Treatment for fibroids and women who are not conceiving	L	Leaves are burnt and the ash mixed with Sodium Bicarbonate which the patient then licks	
Elaeodendron buchananii Loes. (CELASTRACEAE) [MJM 3199]	Omuharanyi	T	A very strong aphrodisiac	R	Roots are boiled with water and the decoction taken as tea or the powder made from dried roots is taken with porridge.	
Eriosema psoraleoides G.Don. Lam. (FABACEAE) [MJM 3196]	Orutanda igwa	S	Malaria (omushana) Aphrodisiac	L, R	Leaves/roots boiled with water	
Flueggea virosa (Willd.) Voigt (EUPHORBIACEAE) [MJM 3203]	Omubwera/omutoruka	S	Gonorrhea and skin conditions	L	Leaves are boiled and decoction administered orally or applied topically.	Saline extract showed antibacterial activity against *Staphylococcus aureus* and *Pasteurella pestis* [24]. Antifungal activity against *Candida albicans and Trichophyton mentagrophytes* [25].
Gutenbergia polycephala Oliv & Hiern (ASTERACEAE) [MJM 3210]	Akatoma	H	To prevent belching of infants so that milk can remain in stomach after breast feeding	L	Leaves are boiled with water and a teaspoonful of decoction given to child as need arises	

Hoslundia opposita Vahl. (LABIATAE) [MJM 3205]	Enzitoima	S	To cleanse the womb of a pregnant woman and to facilitate conception.<br>Used also to ease indigestion	L	For womb cleansing leaves are boiled to make tea while for indigestion they are combined with *Ocimum basillicum*	Used to treat irregular menstruation, and reduce excessive menstrual bleeding [26]
Hygrophylla auriculata(Schum.)Heina (ACANTHACEAE) [MJM 3218]	Kasindano/Karai	H	Malaria, gonorrhea, raise immunity	AP	Aerial parts are boiled and decoction drank	
Jatropha curcas L. (EUPHORBIACEAE) [MJM 3187]	Ekiho	S	Mastitis, mdudu wa kidole	L	Leaves are cooked on fire and sap squeezed out and used to dress affected area daily for 5 days.	Used to treat induration of breast associated with pain and redness. A cataplasm made of leaves from several plant species is applied [27]. Aqueous ethanol extract showed activity against *Staphylococcus aureus* [27, 28].
Kigelia, africana (Lam) Benth (BIGNONIACEAE) [MJM 3202]	Omujunguti	T	Hypertension<br><br>Haematinic	SB<br><br>Pod	The bark is boiled with water and half a cup of the decoction is taken three times a day. A decoction of the pod is also used as haematinic	

Lantana camara L. (VERBENACEAE) [MJM 3213]	Omuuki	S	Gonorrhea, syphilis, swollen legs, cough,and to dilate vagina during labour	R, L	The root/leaves boiled together with stem bark of *Mangifera indica* and *Ocimum basillicum* and decotion taken.	A water extract of the flowers is used for treatment of cough in children and also adults [29, 30]. Decoction of *Lantana camara* and *Salix chinensis* is used as a remedy for rheumatism [30, 31] and has proven anti-inflammatory activity [30]. It is also a remedy for dermatitis, inflammation and skin eruptions [31]. Both antifungal and antibacterial activities have been demonstrated by laboratory results [31].
Maytenus senegalensis Exel Lam. (CELASTRACEAE) [MJM 3197]	Mnyabuliko	S	Burning sensation of the feet, joint pains, skin rashes, weeping rashes	R, SB	Powder made from the roots or stem bark is mixed with powdered roots of *Rauvolfia vomitoria* , *Parinari curatellifolia* and *Ozoroa insignis* subsp. *reticulata* in a fat base. Preparation containing *Rauvolfia vomitoria* should not be applied to open wounds due to its toxicity.	
Melanthera scandens Schumach & Thonn (ASTERACEAE) [MJM 3190]	Kyabakiliaho	H	Ulcers and wounds, lowering blood glucose	L	A thick decoction made using leaves is drank or applied as paste on wounds	Decoction of fresh leaves is used to stop bleeding from cuts, and wound healing. It is used for rapid healing after circumcision [32]
Microglossa pyrifolia Lam. (ASTERACEAE) [MJM 3200]	Omuhe/Mkuraiju	S	Cleansing airways, colds, cough, flu,	L	Leaves are pounded and the sap squeezed into the nostrils.	Decoction of fresh leaves is used for pain in the head, chest and shoulders with chronic rhinitis, for treatment of chronic cough with fever [33, 34], while a decoction of the dried roots is used for treatment of

						colds [34]
Pappea capensis Eckl. Zeyh. (SAPINDACEAE) [MJM 3195]	Omulema mpango	T	Backache	L	Leaves boiled with the leaves of *Vernonia brachycalyx* O. Hoffun and a tablespoonful of the decoction taken three times a day.	
Parinari curatellifolia Plauch Benth. (CHRYSOBALANACEAE) [MJM 3208]	Omunazi	T	Cancers<br>Fungal infection, athlete foot rot, hydrops foetalis<br>Burning sensation of the feet, joint pains, skin rashes, weeping rashes	RB	Cancer: One teaspoonful of powder is taken alone or mixed with honey three times a day until the cancer is treated.<br>Hydrops foetalis: the root bark is boiled with water and the decoction drank<br>Burning sensation of feet: Powder of the roots or stem bark is mixed with root powders of *Rauvolfia vomitoria, Maytenus senegalensis*, and *Ozoroa insignis* subsp. *reticulata* in a fat base. Preparation containing *Rauvolfia vomitoria*.	
Rhus natalensis DC(ANACARDIACEAE) [MJM 3192]	Omushasha	S	Chicken pox	L	A decoction of the leaves is mixed with those of *Vernonia amygdalina* and drank. The leaves are also used for bathing.	
Rhus vulgaris Benth (ANACARDIACEAE) [MJM 3204]	Omukanja	S	Malaria; highly diuretic	L	Leaves are boiled with water and sometimes combined with other plants.	
Rubia cordifolia L. (RUBIACEAE) [MJM 3193]	Karamata	CLH	Warts; reduces excessive menstrual bleeding	AP	Aerial parts are burnt and the ash lick ed or eaten.	Decoction of the entire plant and other parts such as roots and leaves is used for treatment of menstrual disorders such as amenorrhea [35, 36, 37].

Tragia furialis Boj. (EUPHORBIACEAE) [MJM 3186]	Mgonampili	CLH	Hernia, aphrodisiac and backache	R	Root powder is mixed with that of *Elaeodendron buchanannii* Loes (Celestraceae) or *Spathodea campanulata* P. Beaov. (Bignoniaceae) and *Carisa spinarum* L. Mantas (Apocynaceae) and taken with water or boiled and decoction drank	
Trema orientalis L. (ULMACEAE) [MJM 3212]	Omuuwe	T	Yellow fever, haematinic	AP	Leaves are pounded and boiled with the leaves of *Combretum collinum* (Combretaceae) and *Erythrina abbyssinica* (Fabaceae), and decoction used to treat yellow fever. An infusion of the leaves is drank as a haematinic	
Vernonia amygdalina Del. (ASTERACEAE) [MJM 3209]	Omubilizi	T	Febrile convulsions, fever, malaria and mastitis in cows	L, R	Leaves are squeezed the sap that comes out is administered. For treating mastitis in cattle the leaves are pounded and put in drinking water. For malaria and febrile convulsions a decoction made with the stem bark of *Rhus natalensis* (Anacardiaceae),and leaves of *Dalbergia nitidula*, *Desmodium salicifolium* (Poir) DC and *Eriosema psoraleoides*.	Fresh entire plant used to treat fever, and malaria and has proven antimalarial activity [32, 38, 39].
